# A CASE REPORT: RESPIRATORY MANIFESTATIONS OF COVID-19 STARTING WITH A GASTROINTESTINAL COMPLAINT: A COINCIDENCE OR A CORRELATION?

**DOI:** 10.21010/ajidv15i2.4

**Published:** 2021-09-01

**Authors:** Anna Surgean Veterini, Lucky Andriyanto, Hamzah Hamzah

**Affiliations:** † Anesthesiology and Intensive Care, Special Hospital for Infection-Universitas Airlangga, Indonasia

**Keywords:** COVID-19, Gastrointestinal complaints, Abdominal pain

## Abstract

**Background:**

SARS COV-2 is the cause of the current outbreak of COVID-19. The infection of SARS COV-2 causes changes in the gut-lung axis and the intestinal microbiota pro-inflammatory cytokines interaction which leads to the injury of the gastrointestinal tract. One of the symptoms of COVID-19 outside the respiratory system is a complaint in the GIT.

**Materials and Methods:**

We present a COVID-19 case report that begins with a complaint of abdominal pain.

**Results:**

There was no previous suspicion of COVID-19, but after a radiological examination and SARS-COV2 positive PCR result, the patient was proven to be suffering from COVID-19.

**Conclusion:**

After hospitalization in the ICU for about 14 days, a recovery occurred and the patient was able to go home in a very good clinical condition.

## Introduction

COVID-19 often presents a wide variety of gastrointestinal symptoms, such as loss of appetite, nausea, vomiting, diarrhea, and generalized abdominal pain. Previous data showed 18% of patients showing such symptoms, while 16% may only present with gastrointestinal symptoms. In Wuhan, the incidence of gastrointestinal symptoms was 79.1%, and diarrhea was 49.5% among the 305 COVID-19 patients (Tian *et al.*, 2020). There were 5.2% positive fecal microscopic leukocytes among them, and no red blood cells matching the criteria of viral diarrhea were identified. The incidence of nausea was 29.4%, and vomiting was 15.9%, although abdominal pain incidence was relatively low at 6.0% (Ye *et al.*, 2020). The overall prevalence of GIT symptoms in COVID-19 is 18% with the most common symptoms were diarrhea (13%), followed by nausea or vomiting (10%), and abdominal pain (Cheung *et al.*, 2020). Rarely, COVID-19 patients can only present with GI symptoms without respiratory symptoms (Ramachandran *et al.*, 2020). However, most patients were treated with antibiotics (arbidol and ribavirin which is an antiviral agent used for some viral infectious diseases) and nonsteroidal anti-inflammatory drugs (NSAIDs), so drug-associated GIT symptoms should be distinguished (Zhao *et al.*, 2020).

Based on the preceding factors, we would like to present an example of a case report for a COVID-19 patient who had a GIT complaint prior to being diagnosed with COVID-19. The purpose of this study was to provide a discourse that in the mid of the COVID-19 pandemic, various complaints can be mistaken for COVID-19 symptoms as it has a high risk of death.

### Case Presentation

Mr. Y, a 60-year-old household appliance dealer, weighing 65 kg, and 165 cm tall, came to the ER with dizziness, nausea, vomiting, and abdominal pain for 5 days. The patient was previously managed in private hospital for a week where a diagnosis of GIT disorder was made. Before coming to Special Hospital for Infection Universitas Airlangga (Rumah Sakit Khusus Infeksi/RSKI, Universitas Airlangga), this patient experienced complaints of shortness of breath, but there was no cough and febrile. Due to the suspicion of COVID-19, PCR for SARS COV-2 was requested, and the result was positive. Patient was, however, referred to RSKI hospital due to lack of bed-space in the diagnosing hospital where he was immediately admitted into the ICU and was managed for 14 days, followed by low care for 7 days.

Therapy in the previous hospital: potassium tablet 2 times daily (3 days), antacid syrup 3 times daily at 15ml (for 3 days), salt capsules 2 x 500 mg (for 3 days), hydroxy-chloroquine, 2 x 200 mg (for 4 days), liver supplement (contain lecithin, silymarin, schizandra, and vitamin E) 2 times daily (for 1 day), hyoscine butylbromide, 3 times daily if needed, intravenous (iv) vitamin C,1000 mg once daily (for 4 days), corticosteroid once daily at 5 mg (for 4 days), acetylcysteine 1 x 5000 mg (for 4 days), proton pump inhibitors, 2 x 40 mg (1 day), midazolam 2 mg/hr (1 day), antiviral 500/24 hour (1 day), Asering^R^ Infusion 500ml/24 hour (1 day), Clinimix15E^R^ 500ml/24 hour (7 days), plasma convalescent 2 x 200 ml.

Therapy while in RSKI: including antivirals (75 mg two times daily for 7 days), chloroquine 300 mg two times daily for 5 days, probiotics lactobacillus strain three times daily, multivitamin once daily, Curcuma three times daily, heparin 500 IU/hour for eight days, corticosteroid for extra two days, and N-acetyl cysteine, 5000 mg once daily.

Patient was intubated on arrival at the RSKI hospital and mechanically ventilated in the ICU for 15 days. He was extubated with the PaO_2_/FiO_2_ ratio 353, as shown in Table 1. Enteral nutrition was provided with the concept of start low, go slow, 30 Cal/kg body weight/24 hours, divided into 6 times of administration through a nasogastric tube. The nutritional composition consists of 70% carbohydrates, 30% fat of the total calories needed. Protein was given 0.8 grams/kg body weight/day outside of calculating the total calorie requirement.

**Table 1:** Setting mechanical ventilation and blood gas analysis results

MODE			Bilevel	Bilevel	Bilevel	Bilevel	Bilevel	Bilevel	Bilevel	Bilevel			PSV	Nasal cannula
P high/P control			28	28	28	28	28	28	28	28			26	
P support			18	18	18	18	18	18	18	18			18	
RR			24	24	24	24	24	24	24	24			20	
FiO2			40	40	80	80	50	50	40	40			35	4lpm
T inspiration			1:1.7	1:1.7	1:1.7	1:1.7	1:1.7	1:1.7	1:1.7	1:1.7				
PEEP			8	8	8	8	8	8	8	8			8	
pH	7.26	7.4	7.4	7.39	7.35	7.32	7.3	7.37	7.46	7.19	7.46	7.39	7.46	7.43
PO2	94.4	77.7	69	66.4	112.2	125	76.3	80.9	80.7	69.3	85.5	143.5	123.6	74.3
pCO2/Et CO2	56.2	38.2	41.8	39.6	47.4	49.3	62.8	46	36.2	92.5	35.5	24.8	32.6	31.8
HCO3	24.6	24.7	24.9	23.5	25.9	25	30.7	26	25.4	34.7	25.1	14.9	22.7	21
BE	-3.4	0.6	-0.1	-1.3	-0.2	-1.7	2.6	0.3	2	3.1	1.9	-7.8	-0.3	-2.1
SaO2	95.7	95.7	93.4	92.6	98.1	98	93.7	95	96.6	88.6	97.2	143.5	99	95.2
AaDO2	551.9	306.3	239.5	172	404.7	390	137	151	162.4	110.8	158.3	112.3	87.9	95.4
PaO2/FiO2 ratio			172.5	166	140.3	156	152.6	161.8	201.7	173.3	212	358	353.1	353

Sugar containing fruit juice was also provided but the sugar contained therein was not included in calculating the total calorie requirement. The fruit juice was given based on fiber requirements and possible antioxidant content.

The fluid balance was adjusted with a maximum deficit target of 1000 ml/day regarding fluid requirements. All fluids that enter the patient’s body were counted, either intravenously or through a nasogastric tube. Likewise, the discharge fluid, urine, and gastric retention were calculated through the nasogastric tube.

**Figure 1:**
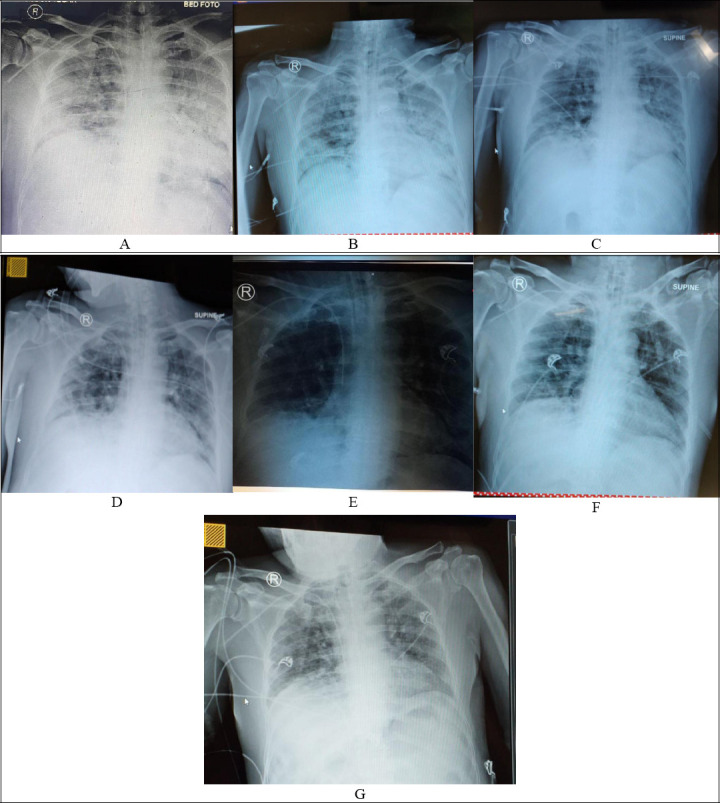
Serial Chest X-ray. A. Oct 6^th^, 2020; B. Oct 7^th^, 2020; C. Oct 10^th^, 2020; D. Oct 13^th^, 2020; E. Oct 16^th^, 2020; F. Oct 19^th^, 2020; G. Oct 22^td^, 2020.

Laboratory tests (Table 2) and chest X-rays (Figure 1) were carried out every 3 days, as shown in Table 1. The antibiotic, tigecycline 50 mg iv was given twice a day to treat *Acinetobacter baumanii* detected on day 8 at RSKI. *Acinetobacter baumanii* infection is known from an increase in leukocytes, evidence of sputum culture, and clinical patients with febrile condition.

**Table 2:** Routine laboratory examination results

	Oct 7, 20	Oct 10, 20	Oct 13, 20	Oct 14, 20	Oct 16, 20	Oct 18, 20	Oct 21, 20
Hemoglobin	12.4	13.1	12.6		13.4	12.4	12.6
Erythrocytes	4.19	4.49	4.3		4.51	4.14	4.24
White Blood Count	16.04	11.94	23.26		26.26	24.45	11.66
Platelet	256	330	320		324	227	220
Hematocrit	37.1	38.9	38.2		39.4	36.3	37.1
Lymphocytes	1.2	2.8	2.4		3.8	2.7	5.6
Neutrophils	93.3	87.1	89		88.9	89.6	80.4
Neutrophil	77.75	31.1	37.08		23.39	33.18	14.36
Lymphocyte ratio							
BUN	16.2	15.5	16.6		24	29.6	21.2
Serum Creatinine	1.09	0.74	0.7	0.8	0.64	0.5
SGOT	74						
SGPT	78						
Bilirubin D/I	0.6/0.27						
Albumin	3.76	3.3	34.6		3.8	3.83	3.61
Sodium	140	135	136		136	136	132
Potassium	4.6	4.3	4.6		4.2	4.1	3.8
Chloride	107	103	107		100	99	100
PTT/Control			12.2/12			13.9/12	15/12
APTT/Control			32/31			25.6/31.5	31.2/31.5
D-Dimer				1.56			2.12
Ferritin							1389
CRP quantitative				10.59			74.54
PCT	0.29			<0.05			0.07

## Discussion

There is need for physicians to be vigilant in the pandemic era and not ignore early symptoms or mild GI symptoms as one of the clinical symptoms of COVID-19. Patients with COVID-19 can develop various GI manifestations, which may or not be accompanied by respiratory symptoms (Galanopoulos *et al.*, 2020). Some of the COVID-19 cases with GI complaints have been reported as previous studies have shown that SARS-CoV-2 can be transmitted through feces. Compared with patients without gastrointestinal symptoms, those with gastrointestinal symptoms are more prone to fatigue, cough, and headache (Ye *et al.*, 2020). However, in this report, the patient recovered well, even though it was a complicated case requiring ICU intervention. Delay in screening at previous hospitals before being admitted to RSKI was a good lesson in this case report study.

SARS-CoV-2 also affects the digestive system and liver, not only a manifestation of the respiratory tract. Duct epithelial cells and liver cells express the angiotensin-2-converting- enzyme (ACE2), the main receptor for SARS-CoV-2. Yang and Tu (2020) analyzed 204 COVID-19 patients with complete laboratory, imaging and historical data. They found 103 patients (50%) experienced digestive disorders, such as loss of appetite (81 [79%] out of 103), diarrhoea (35 [34%]), vomiting (4 [4%]), and abdominal pain (2 (2%]). Although most of the patients showed fever or respiratory symptoms, for six patients, only digestive system disorder appeared during the disease (Yang and Tu, 2020). Another study was reported by Saeed *et al*. (2020) that abdominal pain could be the main symptom of COVID-19 without respiratory complaints (Saeed et al., 2020). We cannot confirm which symptom stimulated the clinical appearance of COVID-19 in the patients we faced first. Either the complaint of GIT disorder was the initiation of SARS COV-2 infection or SARS COV-2 infection in the lungs caused infection in another organ such as GIT, or was it just a coincidence. The first rationalization that this GIT complaint was the initial complaint of COVID-19 was the GIT complaint about 7 days before complaining of shortness-of-breath and coughing. The article written by Buscarini et al. (2020) fits perfectly with the one case we faced. The importance of including GI symptoms was stressed among the spectrum of COVID-19 features to allow early diagnosis and appropriate treatment even in patients without respiratory symptoms (Buscarini *et al.*, 2020). A further explanation for GI complaints is the role of ACE-2 as an essential regulator of intestinal inflammation (Hashimoto *et al.*, 2012).

Second rationalization, a study by Ye *et al*. (2020) showed that SARS COV-2 can affect other organs beside the lungs including the gastrointestinal tract since the high expression of ACE-2 has been detected in intestinal epithelial cells, esophagus, and lungs (Gu *et al.*, 2020). The binding of SARS-CoV-2 to ACE-2 in the gastrointestinal tract reduces the level of available receptors, affects the absorption of tryptophan, and ultimately destroys the intestinal flora’s steady-state, one of the causes of gastrointestinal symptoms such as diarrhea (Ye *et al.*, 2020).

The third rationalization that the incidence of GIT complaints and respiratory problems is just a coincidence, in our opinion, is not suitable because in about 50% of cases of COVID-19, the presence of SARS-CoV-2 in stool samples and detection of SARS-CoV-2 in the intestinal mucosa of infected patients suggest that the invasion of enterocytes expressing ACE-2 can cause enteric symptoms, and the GI tract may be an alternative infection pathway (Ng and Tilg, 2020). The interaction between SARS-CoV-2 and ACE-2 can cause diarrhea. Bioinformatics analysis revealed that ACE-2 is highly expressed in alveolar type-II (AT-2) cells in the lungs and gastric and duodenal epithelial glandular cells. SARS-CoV-2 indirectly damages the digestive system through an inflammatory response chain (Musa, 2020). In addition, in other cases, Ebola and Lassa viral infections, it is said that GIT manifestations dominate at the beginning of symptoms (Sharma and Cappell, 2015), (Dongo *et al.*, 2013).

The specific management in our therapy was the administration of antiviral. The core of the care we provided is immune system enhancement with strict nutritional regulation, supplementation, probiotics, fluid balance, and anti-coagulants. Monitoring closely with laboratory and radiological examinations were also carried out as instructions for further treatment. At first, antibiotics were not given because rises in leukocytes levels and temperature were not found. But the decision was reinforced by the results of culture tests, and finally, antibiotics were given. Negative fluid balance was used in order to avoid edema in tissues and organs.

The overall therapy given to these patients may not necessarily apply to other patients. Each patient has its uniqueness, but some things about this patient may be considered a discourse for further therapy. Besides, the GIT complaints that occur in these patients are a lesson that in the early phases of COVID-19, the infection can present with symptoms that are entirely unrelated to the airways. A previous study that supports our opinion is that of Hormati *et al*. (2020).

## Conclusion

There is a possible causal link between GIT complaints and respiration in COVID-19. However, further studies are needed to prove this point.

### Conflicts of Interest

The authors declare that there are no conflicts of interest associated with this study.

**List of Abbreviations:**
GIT: – gastrointestinal tract
R: – Polymerase Chain Reaction
ICU: – Intensive Care Unit
NSAIDs: – nonsteroidal anti-inflammatory drugs
RR: – respiration rate
PEEP: – Positive end-expiratory pressure
PH: – Power of Hydrogen
BE: – Base excess
PSV: – pressure support ventilation
BUN: – Blood Urea Nitrogen
SGOT: – Serum Glutamic Oxaloasetic Transaminase
SGPT: – Serum Glutamic Pyruvic Transaminase
PTT: – Partial Thromboplastin Time
APTT: – Activated Partial Thromboplastin Time
CRP: – C-Reactive Protein
PCT: – Procalcitonin
AT2: – alveolar type II
